# Activity of Oxantel Pamoate Monotherapy and Combination Chemotherapy against *Trichuris muris* and Hookworms: Revival of an Old Drug

**DOI:** 10.1371/journal.pntd.0002119

**Published:** 2013-03-21

**Authors:** Jennifer Keiser, Lucienne Tritten, Angelika Silbereisen, Benjamin Speich, Roberto Adelfio, Mireille Vargas

**Affiliations:** 1 Department of Medical Parasitology and Infection Biology, Swiss Tropical and Public Health Institute, Basel, Switzerland; 2 University of Basel, Basel, Switzerland; Queensland Institute for Medical Research, Australia

## Abstract

**Background:**

It is widely recognized that only a handful of drugs are available against soil-transmitted helminthiasis, all of which are characterized by a low efficacy against *Trichuris trichiura*, when administered as single doses. The re-evaluation of old, forgotten drugs is a promising strategy to identify alternative anthelminthic drug candidates or drug combinations.

**Methodology:**

We studied the activity of the veterinary drug oxantel pamoate against *Trichuris muris*, *Ancylostoma ceylanicum* and *Necator americanus in vitro* and *in vivo*. In addition, the dose-effect of oxantel pamoate combined with albendazole, mebendazole, levamisole, pyrantel pamoate and ivermectin was studied against *T. muris in vitro* and additive or synergistic combinations were followed up *in vivo*.

**Principal Findings:**

We calculated an ED_50_ of 4.7 mg/kg for oxantel pamoate against *T. muris* in mice. Combinations of oxantel pamoate with pyrantel pamoate behaved antagonistically *in vitro* (combination index (CI) = 2.53). Oxantel pamoate combined with levamisole, albendazole or ivermectin using ratios based on their ED_50_s revealed antagonistic effects *in vivo* (CI = 1.27, 1.90 and 1.27, respectively). A highly synergistic effect (CI = 0.15) was observed when oxantel pamoate-mebendazole was administered to *T. muris*-infected mice. Oxantel pamoate (10 mg/kg) lacked activity against *Ancylostoma ceylanicum* and *Necator americanus in vivo*.

**Conclusion/Significance:**

Our study confirms the excellent trichuricidal properties of oxantel pamoate. Since the drug lacks activity against hookworms it is necessary to combine oxantel pamoate with a partner drug with anti-hookworm properties. Synergistic effects were observed for oxantel pamoate-mebendazole, hence this combination should be studied in more detail. Since, of the standard drugs, albendazole has the highest efficacy against hookworms, additional investigations on the combination effect of oxantel pamoate-albendazole should be launched.

## Author Summary

The roundworm *Ascaris lumbricoides*, the whipworm *Trichuris trichiura* and the two hookworm species *Ancylostoma duodenale* and *Necator americanus* are responsible for the most common infections worldwide and place more than 5 billion people at risk. To control these infections, at risk populations are treated regularly with anthelminthic drugs, mostly albendazole and mebendazole. Since both drugs have a low therapeutic effect against *T. trichiura*, alternative drugs should be discovered and developed. Possible strategies are to re-evaluate forgotten compounds and to thoroughly study drug combinations. We evaluated the activity of the “old”, veterinary drug oxantel pamoate against *T. muris*, *Ancylostoma ceylanicum* and *Necator americanus in vitro* and *in vivo*. In addition, we studied the activity of oxantel pamoate combinations with the four standard treatments for soil-transmitted helminthiasis. Our results confirm that oxantel pamoate has excellent trichuricidal properties. We show that the drug lacks activity against hookworms. It is therefore necessary to combine oxantel pamoate with an anti-hookworm drug. Synergistic effects were observed with oxantel pamoate-mebendazole in our study. Additional preclinical studies should be launched with oxantel pamoate-mebendazole as well as oxantel pamoate-albendazole, since albendazole is the most widely used and efficacious anti-hookworm drug.

## Introduction

Infections with the three major soil-transmitted helminth (STH) species, *Ascaris lumbricoides*, *Trichuris trichiura* and the hookworms *Necator americanus* and *Ancylostoma duodenale* are among the most common parasitic diseases in areas of rural poverty in developing countries [1]. In regions where soil-transmitted helminthiasis is endemic, preventive chemotherapy, i.e. regular anthelminthic drug administration to all people at risk of morbidity, is one of the key strategies [2]. In 2009 it was estimated that 204 million school-aged children were treated for soil-transmitted helminthiasis [3]. The benzimidazoles, albendazole and mebendazole are the most widely used drugs in preventive chemotherapy programs. At present, two alternative drugs, pyrantel pamoate and levamisole are available but currently have a less prominent role since they require weight-based dosing [4]. Despite their excellent safety profile, these drugs have serious limitations with regard to their efficacy. When delivered as a single dose, as in preventive chemotherapy programs, all four compounds have a limited effect against infections with *T. trichiura* as shown in a recent meta-analysis [5]. In addition, drug resistance is a concern [4]; [6]. Efforts are therefore ongoing to discover and develop the next generation of anthelminthic drugs [7]. Promising strategies to identify potential anthelminthic drug candidates are to assess compounds derived from animal health, to re-evaluate forgotten compounds and to thoroughly study drug combinations [7], [8].

Oxantel is the meta-oxyphenol analog of pyrantel. It was discovered in the early 1970s by Pfizer and showed high activity in *T. muris*-infected mice and *T. vulpis*-infected dogs [9], [10]. Subsequent exploratory clinical trials demonstrated that the drug was safe and effective in the treatment of trichuriasis [11]–[14]. For example, complete cure was observed in 10 *T. trichiura*-infected patients treated with 20 mg/kg oxantel pamoate [11]. In veterinary medicine oxantel pamoate was later combined with pyrantel pamoate, which has, with the exception of activity against *Trichuris* spp., a broad spectrum of activity against different nematodes [15]. Today oxantel-pyrantel is widely available as a dewormer for dogs and cats. The combination of oxantel-pyrantel was also evaluated in a few clinical trials against human STH infections [13], . For example, a decade ago oxantel-pyrantel (10 mg/kg) was tested in school-aged children on Pemba island. The combination achieved cure rates of 38.2% and 12.7% against infections with *T. trichiura* and hookworms, respectively [19]. To our knowledge, despite the interesting trichuricidal properties of oxantel, combinations of this drug with other recommended anthelminthic drugs have not been evaluated to date.

The aim of the present study was to investigate the trichuricidal potential of oxantel pamoate combined with the four WHO recommended anthelminthic drugs for the treatment of hookworm, *T. trichiura* and *A. lumbricoides* infections (albendazole, mebendazole, levamisole or pyrantel pamoate) as well as combinations of ivermectin and oxantel pamoate. Ivermectin, the first line drug for strongyloidiasis, is known to have trichuricidal properties and combinations of albendazole-ivermectin and mebendazole-ivermectin have been tested clinically [20]. In a first step the EC_50_ (ED_50_) values of oxantel pamoate against *T. muris* were determined *in vitro* and *in vivo*. Next to oral administration we also tested the activity of intraperitoneal oxantel pamoate in mice. We then elucidated whether oxantel pamoate combined with albendazole, mebendazole, ivermectin, levamisole or pyrantel pamoate interacts in an additive, antagonistic or synergistic manner *in vitro* using the combination index equation [21]. Additive and synergistic combinations were followed up *in vivo*. In addition, the activity of oxantel pamoate was studied against *A. ceylanicum* and *N. americanus in vitro* and *in vivo*.

## Materials and Methods

### Drugs

Albendazole and levamisole were purchased from Fluka (Buchs, Switzerland), oxantel pamoate, mebendazole, ivermectin and pyrantel pamoate were obtained from Sigma-Aldrich (Buchs, Switzerland). Note that, the pamoate salts of oxantel and pyrantel contain only 35.8% and 34.7% of the active ingredients, oxantel and pyrantel base, respectively.

For *in vitro* studies, drug stocks (5–10 mg/ml) were prepared in 100% DMSO (Sigma-Aldrich, Buchs, Switzerland) and stored at 4°C pending usage. For *in vivo* studies, the drugs were suspended in 10% Tween 80 [80% EtOH (70∶30 v/v)] (Buchs, Switzerland) and 90% dH_2_O shortly before treatment.

### Animals

Four week-old female C57BL/10 mice and 3 week-old male Syrian golden hamsters were purchased from Charles River (Blackthorn, UK and Sulzfeld, Germany, respectively). Before infection, animals were allowed to acclimatize for one week in our animal facility. They were kept in groups of maximum ten (mice) or three (hamsters) in macrolon cages with free access to water and rodent food pellets (Rodent Blox from Eberle NAFAG, Gossau, Switzerland).

### Ethics statement

Experiments were performed in an attempt to comply with the 3R rules for animal experiments. The current study was approved by the cantonal veterinary office Basel-Stadt (Switzerland) based on Swiss cantonal and national regulations (permission no. 2070).

### Parasites and infections

#### *Trichuris muris*

The life cycle of *T. muris* has been maintained at the Swiss TPH since January 2010 [22]–[25]. Mice were treated with dexamethasone (1 mg/l, dexamethasone-water soluble, Sigma-Aldrich) supplied with the drinking water 2 days before infection onwards and were infected orally with 200 embryonated *T. muris* eggs.

#### *Ancylostoma ceylanicum* and *Necator americanus*

The *A. ceylanicum* and *N. americanus* life cycles have been maintained at the Swiss TPH since June 2009 and April 2011, respectively, as described previously [25]–[28]. Hamsters were treated with 0.5 mg/l dexamethasone in the drinking water, 2 days before infection onwards. They were infected orally with 150 L3 (*A. ceylanicum*) or subcutaneously with 250 L3 (*N. americanus*). Hamsters assigned to *in vivo* studies were not immunosuppressed and were infected with 300 L3.

### *In vitro* studies with *Trichuris muris*

#### Oxantel monotherapy

Fourth-stage larvae (L4) (days 26–28 p.i.) were collected from the mice intestines (binocular, magnification 16×) and transferred in groups of 3–4 into each well of a 96-well plate containing 100 µl pre-warmed RPMI medium [10.44 g RPMI 1640 (Gibco, Basel, Switzerland), 5 g albumax H (Gibco), 5.94 g HEPES (Sigma-Aldrich) and 2.1 g sodium bicarbonate (Sigma-Aldrich) in 1 l dH_2_O] supplemented with 5% v/v amphotericin B (250 µg/ml, Sigma-Aldrich) and 1% v/v penicillin-streptomycin (10'000 U/ml penicillin+10 mg/ml streptomycin, Sigma-Aldrich). Next, 100 µl of an oxantel pamoate solution were added to obtain 0.15–600 µg/ml (final concentrations) and the plate was incubated at 37°C and 5% CO_2_ for 72 hours. Control worms were incubated in medium with the highest DMSO concentration used in the test (1% v/v). After 24, 48 and 72 hours of incubation the viability of the worms was evaluated according to a motility scale from 3 to 0 (3 = normal, 100% motility, 0 = dead). Assays were conducted in duplicate.

#### Combination chemotherapy studies

Drug combination assays were carried out as described for single drug assays, with slight alterations. Three to 4 adult worms were transferred into each well of a 48-well plate containing 500 µl pre-warmed supplemented RPMI medium. Then, 250 µl of the drug solution #1 and 250 µl of the drug solution #2 were added at a constant dose ratio based on the calculated IC_50_ values (inhibitory concentration 50%) and 2-fold dilutions were carried out up and down. In more detail, the following combinations were tested: 2IC_50_∶2IC_50_, IC_50_∶IC_50_, 0.5IC_50_∶0.5IC_50_ and 0.25IC_50_∶0.25IC_50_. Since for albendazole, mebendazole and ivermectin no IC_50_ value could be calculated (IC_50_s>200 µg/ml) [29], a concentration of 400 µg/ml was selected as IC_50_ value. A combination index (CI) was calculated to characterize the interaction of each combination: synergism (CI<1), antagonism (CI>1) and additive effect (CI = 1) [21].

### *In vitro* studies with *Ancylostoma ceylanicum* and *Necator americanus*

#### Oxantel monotherapy

*In vitro* studies with *A. ceylanicum* and *N. americanus* third-stage larvae (L3) and adult worms were conducted as described recently [25]. Briefly, in a 96-well plate (Costar), 30 L3 per well were incubated for 72 hours at room-temperature in 200 µl HBSS medium supplemented with 10% v/v amphotericin B (250 µg/ml, Sigma-Aldrich), 1% v/v penicillin-streptomycin (10'000 U/ml penicillin+10 mg/ml streptomycin, Sigma-Aldrich) containing oxantel pamoate dilutions (1, 10 and 100 µg/ml, final concentrations). The larval survival was determined microscopically (magnification 20×) following addition of hot water (∼80°C) and exposure to microscope light.

Two to 3 adult worms, collected from the hamsters intestines (binocular, magnification 16×), were incubated per well in 48-well plates for 72 hours in 1 ml supplemented HBSS medium and 10% v/v fetal calf serum containing oxantel pamoate dilutions (ranging from 0.1 to 100 µg/ml) at 37°C, 5% CO_2_. The motility was determined microscopically (magnification 20×) using a viability scale ranging from 2 (normal viability, 100% motility) to 0 (death). Control worms were incubated with the highest DMSO concentration used in the test (2% v/v). Assays were conducted in triplicate.

### *In vivo* studies

#### *Trichuris muris*

Each animal was checked for the presence of eggs in the stools on day 40 p.i. and assigned to treatment or control groups (n = 4 mice per group) and treated with a single oral drug dose on the following day. Oxantel pamoate was administered at 10 mg/kg, 5 mg/kg, 2.5 mg/kg and 1 mg/kg. Two groups of mice were treated intraperitoneally with 10 mg/kg oxantel pamoate and 10 mg/kg ivermectin. Expelled worms, recovered from stools collected for up to 72 hours after treatment, were counted. At dissection, worms remaining in the gut 7 days posttreatment were collected and counted. Worm burden arithmetic means were calculated for each treatment and control group. Worm burden reductions (WBRs) and worm expulsion rates (WERs) were calculated as described previously [25]. Drug combinations revealing synergism *in vitro* (CI<1) (oxantel pamoate-albendazole, oxantel pamoate-levamisole, oxantel pamoate-mebendazole and oxantel pamoate-ivermectin), were tested *in vivo* using a constant dose ratio. The ratio of the ED_50_s (effective dose 50%) of each drug was chosen as starting dose (ED_50_∶ED_50_). If the treatment reduced the worm burden by more than 75% (threshold for additivity when the dose effect curves for both drugs are hyperbolic [21]), the drug doses were divided in half. ED_50_ values of the partner drugs were 345 mg/kg for albendazole, 79 mg/kg for mebendazole (both values determined in the frame of the present work), 4 mg/kg for ivermectin, and 46 mg/kg levamisole [29].

#### *Ancylostoma ceylanicum* and *Necator americanus*

The experiments were carried out as described recently [25], [30]. Briefly, the fecal egg burden was established on days 21 and 22 p.i. (*A. ceylanicum*) and 46 and 47 (*N. americanus*) and treatment and control groups formed on the basis of arithmetic mean fecal egg burden. Hamsters were treated with a single oral dose of 10 mg/kg oxantel pamoate on the following day. Animals left untreated served as controls. The complete stools were collected from each hamster for up to 48 hours posttreatment and searched for expelled worms (binocular, magnification 16×). WERs were calculated.

### Statistical analyses

All the data obtained were analyzed by Excel (Microsoft Office, 2007). *In vitro* data obtained from the individual motility assays were averaged and normalized to the controls. IC_50_s (median-effect dose), defined as the concentration of a drug required to decrease the mean worm's motility to 50% at the 72 hour time point, were calculated with the CompuSyn software (CompuSyn, version 3.0.1). The combination index (CI) was calculated for the combination chemotherapy data with CompuSyn. To test the significance of the WBRs *in vivo*, the Kruskal-Wallis (several treatment doses vs. controls) or the Mann-Whitney U test (one treatment dose vs. control) was applied, using StatsDirect (version 2.4.5; StatsDirect Ltd; Cheshire, UK).

## Results

### *In vitro* studies with *T. muris*

#### Oxantel monotherapy

**Figure 1:**
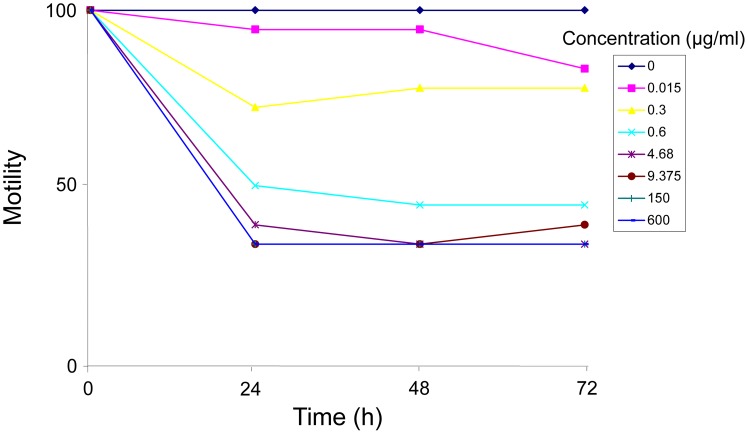
Temporal effect of different concentrations of oxantel pamoate on the viability of *T. muris*. *T. muris* were exposed concentrations of 0.15–600 µg/ml oxantel pamoate and examined 24, 48 and 72 hours post-incubation. Data derived from two independent experiments.

Temporal drug effects of different oxantel pamoate concentrations over the incubation period of 72 hours are depicted in Figure 1. Exposure of *T. muris* L4 to 0.15 and 0.3 µg/ml oxantel pamoate achieved only a negligible effect (mean motilities of 76.7% (SD ±31.3%) and 83.3% (SD ±23.3%), respectively) on the worms 24–72 hours posttreatment. Incubation of *T. muris* L4 for 24–72 hours with 0.6–600 µg/ml oxantel pamoate resulted in strongly reduced viabilities within 24 hours but did not kill the worms. Control worms showed normal movements over the entire incubation period. We calculated an IC_50_ of 2.35 µg/ml for oxantel pamoate (corresponding to 0.78 µg/ml for the free base oxantel) on *T. muris* L4 (Table 1).

**Table 1:** *In vitro* activity of oxantel pamoate against *T. muris*, *A. ceylanicum* and *N. americanus*.

Drugs			IC_50_ (r)			
	*T. muris* L4		*A. ceylanicum*		*N. americanus*	
	IC_50_ (r)	Combination index (CI) at IC_50_	L3	Adults	L3	Adults
Oxantel pamoate	2.35 (0.68)	–	>100 (n.d.)	>100 (n.d.)	>100 (n.d.)	11.80 (0.89)
Oxantel pamoate-albendazole	159.61 (0.87)	0.37	–	–	–	–
Oxantel pamoate-mebendazole	27.95 (0.87)	0.06	–	–	–	–
Oxantel pamoate-levamisole	2.93 (0.99)	0.46	–	–	–	–
Oxantel pamoate-pyrantel pamoate	67.13 (0.90)	2.53	–	–	–	–
Oxantel pamoate-ivermectin	116.86 (0.94)	0.27	–	–	–	–

IC_50_ median effect dose. r = linear correlation coefficient of the median-effect plot, indicating the goodness of fit. r≥0.85 indicates a satisfactory fit. IC_50_s of albendazole, mebendazole, levamisole, pyrantel pamoate, and ivermectin have been published elsewhere [25]. n.d. = not determined.

#### *Trichuris muris* combination chemotherapy

**Figure 2:**
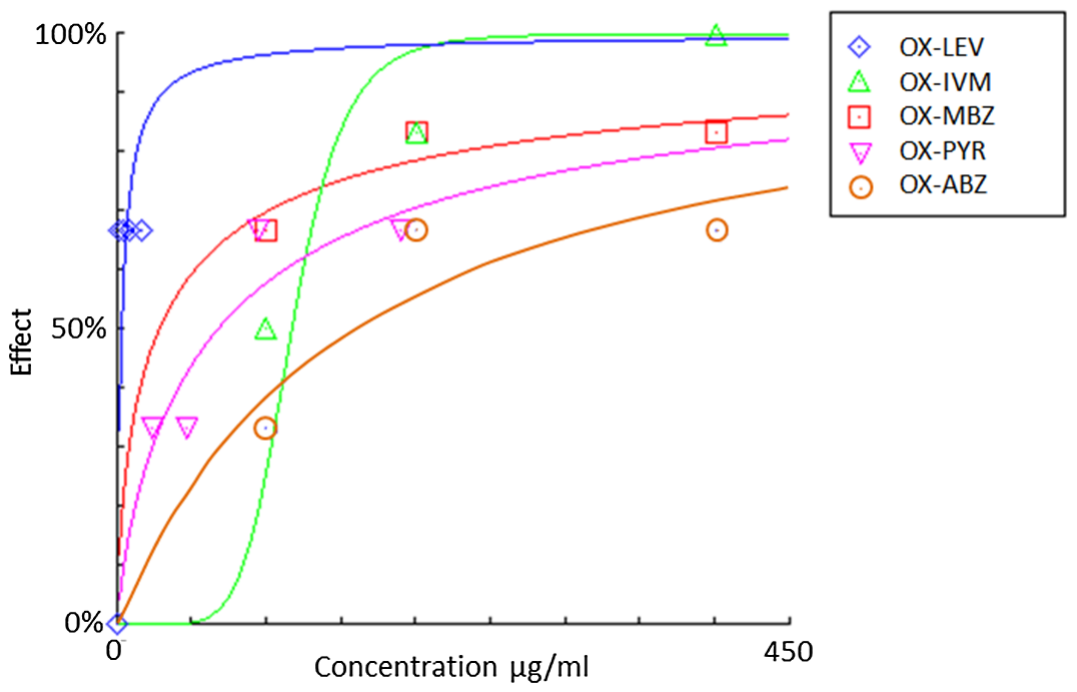
Dose response relationship of oxantel pamoate combinations against *T. muris* in vitro. Oxantel pamoate-levamisole (blue line), oxantel pamoate-ivermectin (green line), oxantel pamoate-mebendazole (red line), oxantel pamoate-pyrantel pamoate (pink line) and oxantel pamoate-albendazole (orange line) were combined using ratios based on their IC_50_s.

Oxantel pamoate was combined with albendazole, mebendazole, pyrantel pamoate, ivermectin or levamisole using ratios based on their IC_50_s and *T. muris* adults were exposed simultaneously to one of these combinations. The results are presented in Table 1 and dose response relationships of the combinations depicted in Figure 2. Synergistic effects were observed for four of the combinations, namely oxantel pamoate-mebendazole (CI = 0.06), oxantel pamoate-ivermectin (CI = 0.27), oxantel pamoate-albendazole (CI = 0.37) and oxantel-pamoate levamisole (CI = 0.46). An antagonistic interaction was found when oxantel pamoate was combined with pyrantel pamoate (CI = 2.53). Worms exposed to this combination were only affected at the two highest concentration ratios (2IC_50_∶2IC_50_ and IC_50_∶IC_50_) and showed normal viability at the two lowest concentration ratios examined (0.5IC_50_∶0.5IC_50_ and 0.25IC_50_∶0.25IC_50_).

#### *In vitro* studies with *Ancylostoma ceylanicum*

*A. ceylanicum* L3 incubated with oxantel pamoate revealed high survival rates (92.9%, SD ±0.01% at 1 µg/ml, 100%, SD ±0.0% at 10 µg/ml and 95.3%, SD ±0.07% at 100 µg/ml), compared to controls. Similarly, adult worms were only weakly affected by the drug, showing an average motility of 100% (SD ±0.0%) at 0.1 and 1 µg/ml and 83.5% (SD ±29.0%) at 10 and 100 µg/ml compared to controls (motility of 100% (SD ±0.0%)).

#### *In vitro* studies with *Necator americanus*

*N. americanus* L3 incubated with oxantel pamoate revealed high survival rates (100%, SD ±0.05% at 0.1 µg/ml, 97.7%, SD ±0.04% at 1 µg/ml, 97.1%, SD ±0.003% at 10 µg/ml and 96.6%, SD ±0.0% at 100 µg/ml), compared to controls. In contrast, adult worms were markedly affected by the drug, resulting in an average motility of 100% (SD ±0.0%) at 0.1 µg/ml, 50% (SD ±25.0%) at 1 µg/ml, 62.5% (SD ±40.5%) at 10 µg/ml and only 12.5% (SD ±25.0%) at 100 µg/ml compared to controls (motility of 100% (SD ±0.0%)). An IC_50_ of 11.80 µg/ml (r = 0.89) was calculated for oxantel pamoate on *N. americanus* adult worms (Table 1).

### *In vivo* studies with *Trichuris muris*

#### Oxantel monotherapy

Oxantel pamoate displayed a high activity against *T. muris in vivo*, with an ED_50_ of 4.71 mg/kg. In more detail, a WBR of 92.5% and WER of 88.4% were achieved after administration of 10 mg/kg (Table 2). Administration of 5 mg/kg resulted in a WBR of 81.1% and a WER of 78.2%. A low activity was observed with oxantel pamoate at 2.5 mg/kg (WER = 24.3%, WBR = 13.5%) and no effect was observed when mice were treated with 1 mg/kg (WER = 1.5%, WBR = 0%). The worm burden in orally oxantel pamoate treated mice was significantly different from untreated mice (*P* = 0.041). An intraperitoneal treatment of 10 mg/kg lacked activity against *T. muris* (both WER and WBR = 0%). For comparison, 10 mg/kg ivermectin given intraperitoneally resulted in a worm burden reduction of 93.5%.

**Table 2:** Activity of oxantel pamoate monotherapy or combination chemotherapy against *T. muris in vivo*.

Group	Dose (mg/kg)	Mean number of worms (SD)	Mean number of expelled worms (SD)	Worm expulsion rate (%)	Worm burden reduction (%)	*P*-value	Combination index (CI)
Control 1	–	157.5 (62.6)	0.2 (0.5)	0.1	–	–	–
Control 2	–	93.3 (9.5)	0.8 (1.0)	0.8	–	–	–
Control 3	–	109.5 (32.9)	0.8 (0.5)	0.7	–	–	–
Control 8	–	56.5 (22.1)	0 (0)	0	–	–	–
Oxantel pamoate	10^1^	91.7 (46.1)	81.0 (44.0)	88.4	92.5	0.041^a^	–
	5^2^	80.3 (40.0)	62.8 (37.5)	78.2	81.1		
	2.5^2^	105.7 (58.5)	25.7 (16.0)	24.3	13.5		
	1^2^	148.3 (94.1)	2.3 (2.1)	1.5	0		
Albendazole	300^3^	69.0 (63.7)	5.8 (4.5)	8.3	41.8	0.293^a^	–
	75^1^	139.0 (54.9)	0.5 (1.0)	0.4	2.6		
Mebendazole	150^2^	99.8 (47.8)	70.3 (36.1)	70.4	68.1	0.006^a^	–
	75^1^	115.8 (36.5)	42.7 (18.6)	36.5	48.3		
Oxantel pamoate	10^8^ i.p.	44.0 (18.7)	0 (0)	0	0	0.857^b^	–
Ivermectin	10^8^ i.p.	80.7 (59.5)	77 (62.0)	95.5	93.5	0.057^b^	–
Control 4	–	123.3 (35.1)	0.3 (0.5)	0.2	–	–	–
Control 5	–	91.3 (23.7)	0 (0)	0	–	–	–
Control 6	–	78.3 (20.8)	0 (0)	0	–	–	–
Control 7	–	94.4 (39.2)	0 (0)	0	–	–	–
Control 8	–	56.5 (22.1)	0 (0)	0	–	–	–
Oxantel pamoate- albendazole	5+345^4^	105.0 (80.1)	76.3 (80.5)	72.6	76.6	0.529^a^	1.90
	2.5+172.5^8^	191.0 (123.4)	44.5 (45.4)	23.3	0		
Oxantel pamoate- mebendazole	5+79^4^	101.3 (39.8)	87.5 (27.6)	86.4	88.8	<0.001^a^	0.15
	2.5+39.5^4^	53.0 (27.8)	41.3 (28.6)	77.8	90.5		
	1.25+19.75^5^	128.8 (68.8)	107.5 (69.3)	83.5	76.7		
	0.63+10^6^	106.8 (49.9)	74.0 (42.7)	69.3	58.2		
Oxantel pamoate- ivermectin	5+4^4^	79.0 (20.8)	60.3 (14.5)	76.3	84.7	0.008^a^	1.27
	2.5+2^5^	116.7 (28.3)	59.7 (15.9)	51.1	37.5		
Oxantel pamoate- levamisole	5+46^7^	60.0 (40.2)	43.0 (30.5)	71.7	82.0	0.028^a^	1.27
	2.5+23^7^	90.0 (45.6)	28.0 (19.4)	31.1	34.3		

Numbers in superscript refer to the corresponding control group.

a *P*-values were obtained from the Kruskal-Wallis test (several treatment doses vs. controls),

b *P*-values were obtained from the Mann-Whitney U test (one treatment dose vs. controls). The CI at IC_50_ are based on WBR.

#### Combination chemotherapy

The four drug combinations that displayed synergistic effects *in vitro* were followed up *in vivo* (Table 2). Simultaneous treatment of *T. muris*-infected mice with a combination of oxantel pamoate and albendazole using the approximate ED_50_ doses resulted in a WBR of 76.6%, while combining 0.5ED_50_s was inefficacious (WBR = 0%). The combination was modeled as antagonistic (CI = 1.90). A synergistic interaction was found for the combination oxantel pamoate-mebendazole, as illustrated by a combination index of 0.15. A WBR of 88.8% was achieved combining both drugs using the ED_50_ doses and a still moderate WBR of 58.2% was observed when doses of 0.63 mg/kg oxantel pamoate and 10 mg/kg mebendazole (1/8 ED_50_s) were administered. Oxantel pamoate combined with ivermectin achieved a WBR of 84.8% at the highest dose tested (ED_50_∶ED_50_ (5 and 4 mg/kg) of oxantel pamoate and ivermectin, respectively), but the combination at 0.5ED_50_∶0.5ED_50_ only produced a worm burden reduction of 37.5%. The combination dose-effect analysis yielded antagonistic properties for the oxantel pamoate-ivermectin combination (CI = 1.27). Finally, while the combination of oxantel pamoate and levamisole at the ED_50_∶ED_50_ removed most of the worms (WBR = 82.0%, WER = 71.7%), using half of the dosage reduced the worm burden by less than 50% (WBR = 34.3%, WER = 31.1%). The overall behavior of the combination of oxantel pamoate and levamisole was found to be antagonistic (CI = 1.27).

#### *In vivo* studies with *A. ceylanicum* and *N. americanus*

Oxantel pamoate exerted no effect on *A. ceylanicum in vivo* following a single dose treatment of 10 mg/kg, illustrated by a WER of 0% (data not shown). The same oral treatment (10 mg/kg) in the *N. americanus* model resulted in a very low WER of 10.3% (data not shown).

## Discussion

Since the introduction of albendazole, mebendazole, levamisole, and pyrantel pamoate in the human armamentarium to treat STH infections 3–4 decades ago, successes in the discovery and development of a novel nematocidal drug have been limited. The danger of resistance development therefore raises concern for the availability of effective therapies in the future. Furthermore, all four above-mentioned drugs have a limited activity against *Trichuris* spp when administered as single oral doses. To accelerate the discovery of novel anthelminthic treatments potential drug candidates have recently been examined *in vitro*, *in vivo* and in clinical trials. Disappointingly, nitazoxanide, a potential drug candidate identified through systematic literature searches [7] as well as a combination of albendazole and nitazoxanide revealed low trichuricidal activity in a randomized placebo controlled trial on Pemba [31]. Furthermore, monepantel, a safe nematocidal drug recently marketed for veterinary use showed a very poor activity against *Ascaris suum* and *T. muris in vitro* and *in vivo* [25]. Hence, neither nitazoxanide nor monepantel can be recommended for the treatment of infections with STH.

In the present work, another potential candidate, oxantel pamoate, widely used in veterinary medicine was evaluated against *T. muris* and hookworms *in vitro* and *in vivo*. Note that one limitation of our study (and helminth drug discovery in general), is that *in vitro* testing relied on motility scoring using microscopy, which is a subjective examination procedure [32].

Oxantel pamoate revealed an excellent trichuricidal activity in mice. We calculated an ED_50_ of 4.7 mg/kg in *T. muris*-infected mice. A similarly low ED_50_ of 1.7 mg/kg was reported previously in this model [33]. For comparison, the WHO recommended drugs for the treatment of STH infections are characterized by much higher ED_50_ values against *T. muris in vivo*, namely 345 mg/kg for albendazole, 79 mg/kg for mebendazole, 46 mg/kg for levamisole and >300 mg/kg for pyrantel pamoate [25], [30]. Ivermectin, used in the treatment of strongyloidiasis and filarial infections, displayed a comparable ED_50_ value of 4 mg/kg in our *T. muris* model [29]. A dose of 10 mg/kg oxantel pamoate administered intraperitoneally lacked activity in *T. muris*-infected mice. For comparison, the same i.p. dose of ivermectin resulted in a high reduction of the worm load (>93%). This demonstrates that in contrast to ivermectin oxantel pamoate does not kill the worm via the blood stream.

Oxantel pamoate lacked *in vivo* activity against both hookworm species *A. ceylanicum* and *N. americanus*. This finding is in line with a previous study in *A. caninum*-infected mice [34]. Interestingly, *N. americanus* adults were affected by the drug *in vitro* while no activity was observed on *A. ceylanicum*. To our knowledge, the activity of oxantel pamoate against hookworms has not been studied in humans.

Oxantel pamoate showed also no effect against the third major soil-transmitted helminth species, *A. lumbricoides* in humans (all 53 patients treated with oxantel revealed *Ascaris* eggs in the stools collected posttreatment regardless of the dose administered) [11]. It is therefore necessary to combine oxantel pamoate with a partner drug with a therapeutic profile that covers roundworms and hookworms. In the present work we have, for the first time, thoroughly evaluated the potential of oxantel pamoate in drug combinations. This work builds on a series of laboratory investigations on the potential of combination chemotherapy for the treatment of STH infections. We have for example recently examined combinations of marketed drugs in *in vitro* and *in vivo* studies against *T. muris* [8].

Interestingly, antagonistic effects were observed in the present work with oxantel pamoate-pyrantel pamoate against *T. muris in vitro*, hence this combination was not pursued further. However, we cannot exclude a better trichuricidal effect *in vivo* for this combination, in particular as a pharmacodynamic interference at the target is unlikely. Oxantel is classified as an N-subtype AChR agonist, while pyrantel is considered an L-subtype suggesting differences in drug action [35]. The combination of oxantel pamoate-pyrantel pamoate is widely used in veterinary medicine and has also been studied in several human clinical trials. For example, in Korea oxantel pamoate-pyrantel pamoate at 20 mg/kg achieved a cure rate of 75% and egg reduction rate of 97% against *T. trichiura* infections and cleared *A. lumbricoides* infections [13]. A high egg reduction rate against *T. trichiura* following oxantel pamoate-pyrantel pamoate at 20 mg/kg was also reported in a Malaysian study [18]. A lower effect of this combination administered at 10 mg/kg was observed on Pemba with cure rates of 96.3, 38.2 and 12.7% against *A. lumbricoides*, *T. trichiura* and hookworm, respectively [19]. In two Korean trials both oxantel monotherapy as well as an oxantel-pyrantel combination were used, however since different formulations were used (syrup versus tablets), different dosages applied and sample sizes were small no conclusion can be drawn whether the combination was superior to oxantel monotherapy [13], [14].

Antagonistic effects were observed *in vivo* using combinations of oxantel pamoate-albendazole, oxantel pamoate-levamisole and oxantel pamoate-ivermectin. Since the molecular basis for the actions of these drugs is not yet fully elucidated it is impossible to explain the antagonistic interaction profile observed for these combinations. On the other hand, the oxantel pamoate-mebendazole combination revealed highly synergistic effects against *T. muris in vivo*. It is striking that the two benzimidazole derivates behave so differently when administered as partner drugs in oxantel pamoate combinations to *T. muris* infected mice given that both drugs, despite their differences in pharmacokinetics [36], have identical targets. However, our results should be interpreted with caution. First of all, drug scheduling and drug vehicle, solubility, host behavior, environmental factors and genetic variations might influence the level of activity [37]. In addition, though the median effect method used in the present work is the most commonly used, our data are based on a single method only and one could have considered applying another method, such as the isobologram method to re-analyze the data [38]. Finally, note that these findings are based on a single ratio of the combined agents (ED_50_ values) and it might be worthwhile to assess other ratios of the drug dosages.

In conclusion, our study confirms that oxantel pamoate has excellent trichuricidal properties. In the *T. muris* mouse model oxantel pamoate showed a higher activity than the standard drugs albendazole, mebendazole, levamisole and pyrantel pamoate. Since the drug has no activity against hookworms it is necessary to combine oxantel pamoate with a partner drug revealing anti-hookworm properties. Synergistic effects were observed for oxantel pamoate-mebendazole. Despite of our results pointing to an antagonistic behavior of oxantel pamoate-albendazole additional investigations on the effect of this combination might be considered (e.g. evaluation of a different dosing ratio or schedule) since of the standard drugs albendazole has the highest efficacy against hookworms [5]. Systemic drug interactions between oxantel pamoate and partner drugs are unlikely given that the absorption of oxantel pamoate is very poor [11]. Nonetheless, preclinical studies should carefully elucidate metabolic and pharmacokinetic interactions of oxantel pamoate and the benzimidazoles.
